# New Developments in Scheduling Applications

**DOI:** 10.1155/2014/268941

**Published:** 2014-04-28

**Authors:** M. Duran Toksarı, Daniel Oron, Emel Kızılkaya Aydoğan, Jorge G. Barbosa

**Affiliations:** ^1^Department of Industrial Engineering, Erciyes University, 38039 Kayseri, Turkey; ^2^Business School, The University of Sydney, NSW 2006, Australia; ^3^Universidade do Porto, Faculdade de Engenharia, Departamento de Engenharia Informática, Laboratório de Inteligência Artificial e Ciência dos Computadores, Rua Dr. Roberto Frias, 4200-465, Portugal

Scheduling problems are widely recognized as important optimization problems. Thus, scheduling theory has become a fundamental area within the general field of combinatorial optimization. Multiprocessor and shop scheduling problems are known to be hard to solve optimally. Scheduling problems are naturally very varied, both in application domains and in featured constraints. To date, researchers have been working on scheduling problems derived from new applications such as scheduling problems in logistical airport operations management processes, decentralized systems and selfish organizations, grid computing, and bioinformatics. These scheduling problems reflect real-life situations for including learning effects or deteriorating jobs.

In the call for papers, we advised that all authors should be encouraged to focus on scheduling heuristics, scheduling with some effects such as learning and deterioration, grouping and sequencing operations in multistage systems, scheduling in flexible shops, scheduling under some constraints such as precedence, batching/lot sizing, setups, and further technologies, scheduling under uncertainty, and scheduling in a supply chain. The obtained responses gratified us with a total of 24 submissions. All of them were peer reviewed according to high standards of this journal. At the end of the process, we accepted seven papers. The accepted papers represent excellent work that spans across a wide variety of cutting edge scheduling problems and applications.

D. C. Dietz proposes a study on computing the expected cost of an appointment schedule for statistically identical customers with probabilistic service times. In this paper, the author presented a cogent method to compute the expected cost of an appointment schedule. Customers are statistically identical, the service time distribution has been known as mean and variance, and no-shows occur with time-dependent probability.

X. Shi and D. Xu develop a solution for single-machine scheduling with increasing linear maintenance durations by the best possible approximation algorithms. They used the linear form *f*(*t*) = *a* + *bt* with *a* ≥ 0 and *b* > 1. They proposed an approximation algorithm named FFD-LS2I with a worst-case bound of 2 for problem. Furthermore, they also showed that there is no polynomial time approximation algorithm with a worst-case bound for the problem with *b* ≥ 0 unless *P* = *NP*, which implies that the FFD-LS2I algorithm is the best possible algorithm for the case *b* > 1.

T. Luo and Y. Xu investigate semionline scheduling on two machines with grade of service (GoS) levels and partial information of processing time. They worked on three different semionline versions (knowing total processing time of the jobs with higher GoS level or knowing total processing time of the jobs with lower GoS level or knowing both in advance) and proposed algorithms with competitive ratios to solve these semionline versions.

W. Liu et al. develop a time scheduling model of logistics service supply chain (LSSC) based on the customer order decoupling point. They tested their algorithm using numerical analysis for a specific example and obtained interesting results. The order completion time of the LSSC can be delayed or be ahead of schedule but cannot be infinitely advanced or infinitely delayed. The optimal comprehensive performance can be effective if the expected order completion time is appropriately delayed.

F. Tahriri et al. propose a study on a fuzzy mixed model assembly line sequencing and scheduling using multiobjective dynamic fuzzy genetic algorithm. They proposed a new multiobjective dynamic fuzzy genetic algorithm to solve a fuzzy mixed-model assembly line sequencing problem where the primary goal is to minimize the makespan, setup time, and cost simultaneously. They performed a simulation to compare the proposed novel optimization algorithm and the standard genetic algorithm in mixed assembly line sequencing model. The obtained results highlight that the performance and effectiveness of the proposed novel optimization algorithm are more efficient than the performance of the standard genetic algorithm.

V. Fernandez-Viagas and J. M. Firaminan study integrated project scheduling and staff assignment with controllable processing times. They proposed an integer programming model to solve problem, together with some extensions to cope with different settings. Furthermore, the advantages of the controllable processing times approach are compared with the fixed processing times, and they applied a simple GRASP algorithm due to the complexity of the integrated model.

H. Gong et al. present a parallel-batch scheduling and transportation coordination with waiting time constraints. They solved the parallel-batch scheduling problem that incorporates transportation of raw materials or semifinished products before processing under the consideration of waiting time constraints. Furthermore, they proposed an optimal algorithm in polynomial time to solve the case with equal processing times and equal transportation times for each order.

In summary, the seven papers represent some of the latest and most promising research results on scheduling problems. We believe that they make significant impact on solving both theoretical problems and real life applications. We are confident that this special issue will stimulate further research in this area.

